# Highly Pathogenic Avian Influenza Virus (H5N8) Clade 2.3.4.4 Infection in Migratory Birds, Egypt

**DOI:** 10.3201/eid2306.162056

**Published:** 2017-06

**Authors:** Abdullah A. Selim, Ahmed M. Erfan, Naglaa Hagag, Ali Zanaty, Abdel-Hafez Samir, Mohamed Samy, Ahmed Abdelhalim, Abdel-Satar A. Arafa, Mohamed A. Soliman, Momtaz Shaheen, Essam M. Ibraheem, Ibrahim Mahrous, Mohamed K. Hassan, Mahmoud M. Naguib

**Affiliations:** National Laboratory for Veterinary Quality Control on Poultry Production, Animal Health Research Institute, Giza, Egypt (A.A. Selim, A.M. Erfan, N. Hagag, A. Zanaty, A.-H. Samir, M. Samy, A. Abdelhalim, A.-S.A. Arafa, M.A. Soliman, M. Shaheen, E.M. Ibraheem, M.K. Hassan, M.M. Naguib);; General Organization for Veterinary Services, Giza (I. Mahrous);; Friedrich-Loeffler-Institut, Federal Research Institute for Animal Health, Insel-Riems, Germany (M.M. Naguib)

**Keywords:** highly pathogenic avian influenza, influenza, subtype H5N8, migratory birds, clade 2.3.4.4, Egypt, Africa, viruses, zoonoses

## Abstract

We isolated highly pathogenic avian influenza virus (H5N8) of clade 2.3.4.4 from the common coot (*Fulica atra*) in Egypt, documenting its introduction into Africa through migratory birds. This virus has a close genetic relationship with subtype H5N8 viruses circulating in Europe. Enhanced surveillance to detect newly emerging viruses is warranted.

Avian influenza is a highly contagious disease of poultry that continues to spread across the globe in bird populations. Occasionally, transmission of a highly pathogenic avian influenza virus (HPAIV) from infected poultry to humans results in a severe public health crisis (*1*).

In 2010, strains of HPAIV (H5N8) of clade 2.3.4.4 were first detected among wild birds in Asia and later spread to domestic birds across China, South Korea, and Japan (*2*,*3*). Most recently, a novel reassortant virus of subtype H5N8 clade 2.3.4.4 was reported in Russia and further spread to many countries in Europe, Asia, and the Middle East (*4*,*5*). The spread of HPAIV (H5N8) strains has been linked to the overlapping flyways of migratory wild birds that come from different continents; this mingling of wild birds poses a major concern worldwide (*4*,*6*).

Egypt is one of the most notable migration spots for migratory birds crossing Europe, Asia, and Africa. In early winter each year, thousands of migrating waterfowl use Egypt as a resting stop before they continue their journey southward through the African continent through the East Africa/East Asia and Mediterranean/Black Sea migratory flyways. Lake Manzala in northern Egypt is a source of fish and a major refuge for many migratory birds (Technical Appendix Figure 1).

During a targeted surveillance for avian influenza viruses (AIVs) conducted in migratory birds by Community Animal Health Outreach (CAHO) program on November 24, 2016, we collected 19 oropharyngeal and cloacal swab samples from diseased (mild depression) and dead migratory birds (common coot, *Fulica atra*; pintail ducks, *Anas acuta*; and Garganey ducks, *A. querquedula*) in a live bird and fish market in the Damietta Governorate in Egypt. (Hunted migratory birds are commonly sold for food in markets in this region.) Two samples from 2 common coots were confirmed positive for AIV and were subtyped as H5N8 by using specific real-time reverse transcription quantitative PCR (RT-qPCR) (Technical Appendix). On November 30, 2016, the identification of HPAIV (H5N8) from 2 common coots was reported to the World Organisation for Animal Health. Notably, this newly emerged HPAIV (H5N8) was detected in Egypt in the same place, Damietta Governorate, where HPAIV (H5N1) was first identified in 2006 during the global spread of HPAIV (H5N1) viruses of clade 2.2 (*7*). Immediately thereafter, active targeted surveillance for AIV was conducted around Lake Manzala and the surroundings areas for AIV that included wild birds and domestic poultry; however, no more positive cases were detected.

We successfully isolated and characterized 1 HPAIV (H5N8) strain by nucleotide sequencing and phylogenetic analyses on the basis of its hemagglutinin (HA) and neuraminidase (NA) gene segments. The isolate was named A/common coot/Egypt/CA285/2016 (EG-CA285).

The amino acid sequence of the protease cleavage site of EG-CA285 HA protein revealed multiple basic amino acids, PLREKRRKR/GLF, which is characteristic of HPAIV. The receptor-binding pocket of EG-CA285 HA protein showed markers of avian receptor–specific binding: Q222 and G224. We observed 3 amino acid assignment differences in the HA protein, namely, R22K, E268G, and D487Y, which distinguished EG-CA285 from the recent HPAIV (H5N8) clade 2.3.4.4b strain isolated in Russia (A/great-crested-grebe/Uvs-Nuur-Lake/341/2016; GISAID accession no EPI_ISL_224580) (Figure, panel A). In the NA protein, we observed 4 substitution mutations (V8A, V31L, G126E, I407T) that distinguished the EG-CA285 from the subtype found in Russia. Phylogenetic analysis of HA and NA gene sequences revealed that EG-CA285 virus is clustered with clade 2.3.4.4b, along with the recent viruses widely distributed throughout Europe (Figure, panel B; Technical Appendix Figure 2). Even though the unavailability of a full-length genomic sequence of this virus is a limitation in this study, the genetic and phylogenetic features of the HA and NA gene segments confirm the intercontinental dissemination of HPAIV (H5N8) through wild birds and its introduction into Egypt.

**Figure:**
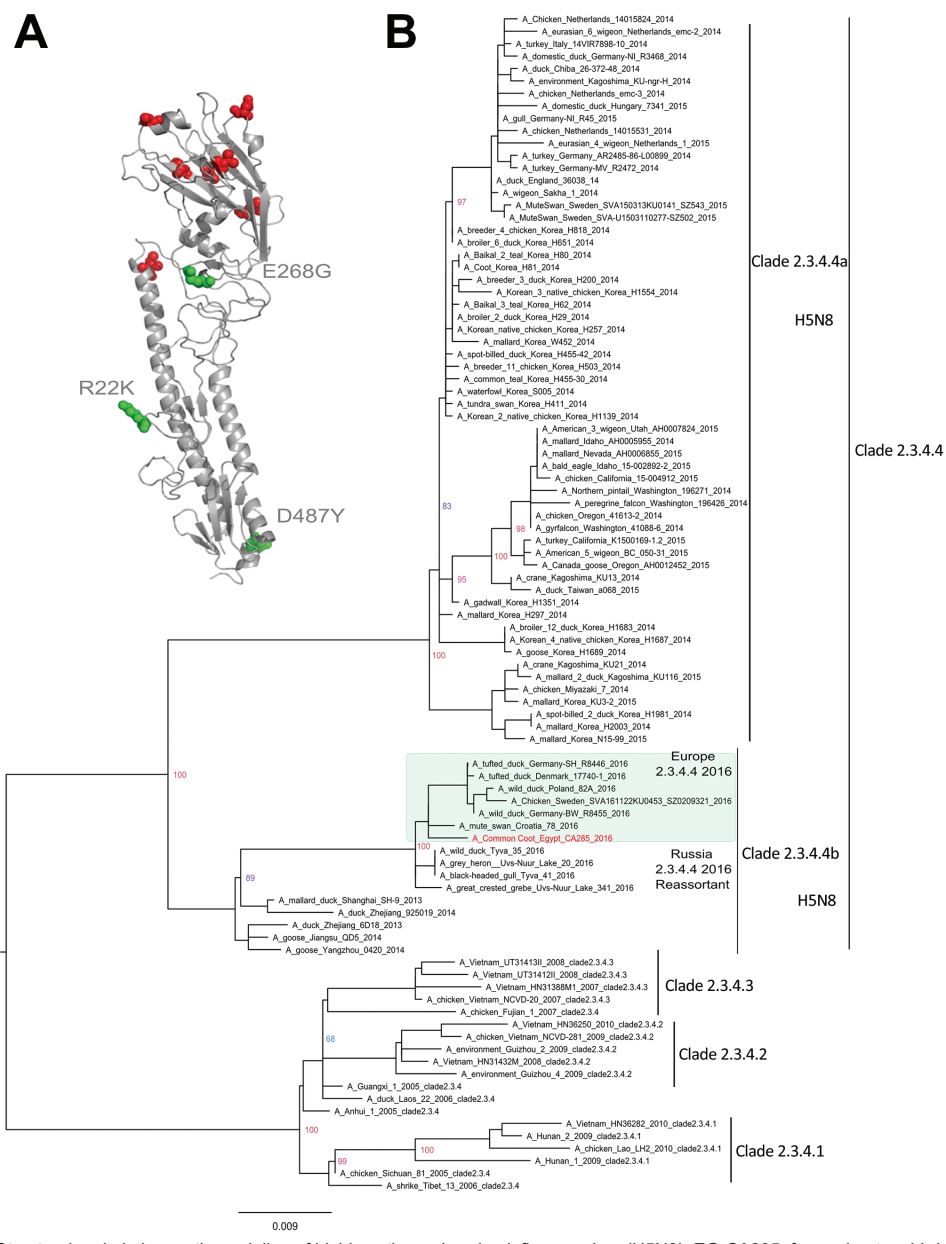
Structural and phylogenetic modeling of highly pathogenic avian influenza virus (H5N8), EG-CA285, from migratory birds, Egypt, 2016. A) Three-dimensional structural homology model for the hemagglutinin protein of EG-CA285 created by using the ancestral virus of clade 2.3.4.4b (A/duck/Zhejiang/6D18/2013 [H5N8]) as a template. Amino acids distinguishing the EG-CA285 sequence from the modeling template are shown in red; green depicts unique mutations distinguishing this virus from the virus detected in summer 2016 in Russia, A/great crested grebe/Uvs-Nuur-Lake/341/2016. B) Phylogenetic tree of the nucleotide sequences of avian influenza virus hemagglutinin genes. Maximum-likelihood calculations were done with IQ-TREE software (http://iqtree.cibiv.univie.ac.at/) under the best-fit model according to the Akaike criterion (general time reversible plus gamma plus G4 model). Red indicates strains from Egypt; green shading indicates strains currently circulating in Europe. Scale bar indicates nucleotide substitutions per site.

During the evolution of subtype H5Nx viruses of clade 2.3.4.4, frequent reassortment has been noted with other co-circulating HPAIVs and low pathogenicity AIVs in different countries in Europe, North America, and East Asia (*8*). Strains of HPAIV (H5N8) have been involved in multiple independent reassortment events with other AIV subtypes found in wild birds in China, South Korea, the United States, and recently in Russia (*5*,*9*). The probable introduction of HPAIV (H5N8) to poultry populations in Egypt will further complicate disease control and prevention, especially if HPAIV (H5N1) of clade 2.2.1.2 and low pathogenicity AIV (H9N2) strains of G1 lineage are enzootic in poultry (*10*). 

In addition, the threat of emergence of a novel reassortants with unpredictable gene constellations of HPAIV (H5N8) strains with enzootic strains of AIV is a public health concern. Therefore, we recommend enhanced surveillance to quickly detect newly emerged viruses. Commercial and backyard poultry owners must follow the recommended biosecurity measures. The detection and immediate reporting of novel HPAIV (H5N8) strains in Egypt will help increase AIV surveillance, detection, and prevention preparedness in other countries of continental Africa.

Technical Appendix Additional methods, GISAID submitters for influenza virus segments used, map of migratory flyways crossing Egypt, and phylogenetic tree neuraminidase gene segments for study of highly pathogenic avian influenza virus (H5N8) clade 2.3.4.4 infection in migratory birds, Egypt.
