# Supplementary material for: Highly Pathogenic Avian Influenza Virus (H5N8) Clade 2.3.4.4 Infection in Migratory Birds, Egypt
Source: Emerg Infect Dis. 2017 Jun;23(6):1048–51. doi: 10.3201/eid2306.162056 (PMC5443452; doi:10.3201/eid2306.162056)
Supplement: Technical Appendix — Additional methods, GISAID submitters for influenza virus segments used, map of migratory flyways crossing Egypt, and phylogenetic tree neuraminidase gene segments for study of highly pathogenic avian influenza virus (H5N8) clade 2.3.4.4 infection in migratory birds, Egypt. [file 16-2056-Techapp-s1.pdf]

Article DOI: http://dx.doi.org/10.3201/eid2306.162056

Highly Pathogenic Avian Influenza Virus (H5N8) Clade 2.3.4.4 Infection in Migratory Birds, Egypt

Technical Appendix

Material and Methods

Samples and Virus Isolation

Nineteen swab samples were collected from the common coot (*Fulica atra*), pintail ducks (*Anas acuta*), and Garganey ducks (*Anas querquedula*) in live bird and fish markets in Damietta Governorate in Egypt. Samples were obtained during a targeted active surveillance of wild birds conducted by Community Animal Health Outreach (CAHO) team. Samples were submitted to the National Laboratory of Veterinary Quality Control on Poultry Production (NLQP) for virus identification and isolation. On November 27, 2016, 2 samples from common coots were confirmed positive for avian influenza virus (AIV) and subtyped as H5N8. On November 30, 2016, the identification of AIV(H5N8) from a common coot was reported to the World Organisation for Animal Health (OIE) as the first case of an outbreak in Egypt in Africa. One virus was successfully isolated through allantoic fluid inoculation of 10-day-old specific-pathogen-free (SPF) embryonated chicken eggs according to the OIE diagnostic manual according to standard protocols (*1*).

RNA Extraction and Molecular Diagnosis

Viral RNA was extracted from the obtained samples by using the QIAamp Viral RNA Mini Kit (QIAGEN, Hilden, Germany) according to the manufacturer’s instructions. All samples were tested using standard reverse transcription quantitative PCR (RT-qPCR) for the M gene of influenza A viruses (*2*). Positive AIV RNA was subtyped for H5, H7, and H9 subtypes and neuraminase (NA) subtyping by using specific subtyping RT-qPCR (*3*,*4*).

Sequencing and Phylogenetic Analyses

Complete gene segments of the hemagglutin (HA) and NA were amplified by using primers previously described by Hoper et al. (*5*). The gene-specific RT-PCR amplicons were size-separated by agarose gel electrophoresis, excised and purified from gels by using the QIAquick Gel Extraction Kit (QIAGEN). Further, purified PCR products were used directly for cycle sequencing reactions (BigDye Terminator v3.1 Cycle Sequencing Kit; Applied Biosystems, Foster City, CA, USA). Reaction products were purified by using Centrisep spin column (Thermo Fisher, Carlsbad, CA, USA) and sequenced on an ABI PRISM 3100 Genetic Analyzer (Life Technologies, Carlsbad, CA, USA). Thereafter, the obtained sequences of the HA and NA genes were assembled and edited by using the Geneious software, version 9.0.5 (*6*). A BLAST (blast.ncbi.nlm.nih.gov/) search was performed by using GISAID platform, and sequences established in this study have been submitted to the Global Initiative on Sharing All Influenza Data (GISAID) database (accession nos.: EPI868853–4). In addition, genetic sequences of representative subtypes H5N8 and H5Nx were retrieved from the GISAID platform. Alignment and identity matrix analyses were performed by using MAFFT (*7*) and BioEdit (*8*). Phylogenetic analyses were based on maximum likelihood methodology based on Akaike criterion after selection of the best- fit modes (GTR+Γ+G4 and HKY+G4 for the HA and NA, respectively) by using IQ-TREE software version 1.1.3 (*9*). Trees were finally viewed and edited with FigTree v1.4.2 software (http://tree.bio.ed.ac.uk/software/figtree/).

References

1. World Organisation for Animal Health. Chapter 2.3.4. Avian influenza. [cited 22 Nov 2014]. http://www.oie.int/fileadmin/Home/eng/Health_standards/tahm/2.03.04_AI.pdf 2014.

2. Spackman E, Senne DA, Bulaga LL, Myers TJ, Perdue ML, Garber LP, et al. Development of real-time RT-PCR for the detection of avian influenza virus. Avian Dis. 2003;47(Suppl):1079–82. PubMed http://dx.doi.org/10.1637/0005-2086-47.s3.1079

3. Monne I, Ormelli S, Salviato A, De Battisti C, Bettini F, Salomoni A, et al. Development and validation of a one-step real-time PCR assay for simultaneous detection of subtype H5, H7, and H9 avian influenza viruses. J Clin Microbiol. 2008;46:1769–73. PubMed http://dx.doi.org/10.1128/JCM.02204-07

4. Hoffmann B, Hoffmann D, Henritzi D, Beer M, Harder TC. Riems influenza A typing array (RITA): an RT-qPCR-based low density array for subtyping avian and mammalian influenza a viruses. Sci Rep. 2016;6:27211. PubMed http://dx.doi.org/10.1038/srep27211

5. Höper D, Hoffmann B, Beer M. Simple, sensitive, and swift sequencing of complete H5N1 avian influenza virus genomes. J Clin Microbiol. 2009;47:674–9. PubMed http://dx.doi.org/10.1128/JCM.01028-08

6. Kearse M, Moir R, Wilson A, Stones-Havas S, Cheung M, Sturrock S, et al. Geneious Basic: an integrated and extendable desktop software platform for the organization and analysis of sequence data. Bioinformatics. 2012;28:1647–9. PubMed http://dx.doi.org/10.1093/bioinformatics/bts199

7. Katoh K, Standley DM. MAFFT multiple sequence alignment software version 7: improvements in performance and usability. Mol Biol Evol. 2013;30:772–80. PubMed http://dx.doi.org/10.1093/molbev/mst010

8. Hall T. BioEdit: a user-friendly biological sequence alignment editor and analysis program for Windows 95/98/NT. Nucleic Acids Symp Ser. 1999;41:95–8.

9. Nguyen LT, Schmidt HA, von Haeseler A, Minh BQ. IQ-TREE: a fast and effective stochastic algorithm for estimating maximum likelihood phylogenies. Mol Biol Evol.

Technical Appendix Table. GISAID submitters of influenza virus segments used in this study

Segment ID	Segment	Country	Collection date	Isolate name	Submitting laboratory
EPI573173	NA	Netherlands	2014 Nov 20	A/Chicken/Netherlands/14015824/2014	Central Veterinary Institute
EPI573171	HA	Netherlands	2014 Nov 20	A/Chicken/Netherlands/14015824/2014	Central Veterinary Institute
EPI596301	HA	Netherlands	2014 Nov 21	A/chicken/Netherlands/emc-3/2014	Other database Import
EPI596303	NA	Netherlands	2014 Nov 21	A/chicken/Netherlands/emc-3/2014	Other Database Import
EPI573179	HA	Netherlands	2014 Nov 21	A/duck/Netherlands/14015898/2014	Central Veterinary Institute
EPI573181	NA	Netherlands	2014 Nov 21	A/duck/Netherlands/14015898/2014	Central Veterinary Institute
EPI596295	HA	Netherlands	2014 Nov 24	A/eurasian wigeon/Netherlands/2/2014	Other database import
EPI596297	NA	Netherlands	2014 Nov 24	A/eurasian wigeon/Netherlands/2/2014	Other database import
EPI691836	HA	Germany	2014 Dec 1	A/gull Germany-NI/R45/2015	Friedrich-Loeffler-Institut
EPI691837	NA	Germany	2014 Dec 1	A/gull Germany-NI/R45/2015	Friedrich-Loeffler-Institut
EPI687238	NA	Germany	2014 Dec 15	A/turkey/Germany/AR3390-L00939/2014	Friedrich-Loeffler-Institut
EPI687239	HA	Germany	2014 Dec 15	A/turkey/Germany/AR3390-L00939/2014	Friedrich-Loeffler-Institut
EPI687246	NA	Germany	2014 Dec 15	A/turkey/Germany/AR3382-L00937/2014	Friedrich-Loeffler-Institut
EPI687247	HA	Germany	2014 Dec 15	A/turkey/Germany/AR3382-L00937/2014	Friedrich-Loeffler-Institut
EPI584823	HA	Hungary	2015 Feb 23	A/domestic duck/Hungary/7341/2015	Central Agricultural Office Veterinary Diagnostic Directorate
EPI584825	NA	Hungary	2015 Feb 23	A/domestic duck/Hungary/7341/2015	Central Agricultural Office Veterinary Diagnostic Directorate
EPI576393	NA	Sweden	2015 Mar 5	A/MuteSwan/Sweden/SVA150313KU0141/ SZ543/2015	National Veterinary Institute

Segment ID	Segment	Country	Collection date	Isolate name	Submitting laboratory
EPI576391	HA	Sweden	2015 Mar 5	A/MuteSwan/Sweden/SVA150313KU0141/ SZ543/2015	National Veterinary Institute
EPI823756	HA	Russian Federation	2016 May 25	A/black-headed gull/Tyva/41/2016	WHO National Influenza Centre Russian Federation
EPI823758	NA	Russian Federation	2016 May 25	A/black-headed gull/Tyva/41/2016	WHO National Influenza Centre Russian Federation
EPI836606	HA	Russian Federation	2016 May 25	A/gray heron /Uvs-Nuur Lake/20/2016	Research Institute of Experimental and Clinical Medicine
EPI836608	NA	Russian Federation	2016 May 25	A/gray heron /Uvs-Nuur Lake/20/2016	Research Institute of Experimental and Clinical Medicine
EPI773759	NA	Russian Federation	2016 May 25	A/great crested grebe/Uvs-Nuur Lake/341/2016	Research Institute of Experimental and Clinical Medicine
EPI773757	HA	Russian Federation	2016 May 25	A/great crested grebe/Uvs-Nuur Lake/341/2016	Research Institute of Experimental and Clinical Medicine
EPI823748	HA	Russian Federation	2016-May-25	A/wild duck/Tyva/35/2016	WHO National Influenza Centre Russian Federation
EPI823750	NA	Russian Federation	2016 May 25	A/wild duck/Tyva/35/2016	WHO National Influenza Centre Russian Federation
EPI859649	NA	Germany	2016 Nov 1	A/wild duck/Germany-BW/R8455/2016	Friedrich-Loeffler-Institut
EPI859650	HA	Germany	2016 Nov 1	A/wild duck/Germany-BW/R8455/2016	Friedrich-Loeffler-Institut
EPI860232	NA	Poland	2016 Nov 2	A/wild duck/Poland/82A/2016	National Veterinary Research Institut Poland, PIWet-PIB
EPI860231	HA	Poland	2016 Nov 2	A/wild duck/Poland/82A/2016	National Veterinary Research Institut Poland, PIWet-PIB
EPI859213	NA	Germany	2016 Nov 7	A/tufted_duck/Germany-SH/R8446/2016	Friedrich-Loeffler-Institut
EPI859212	HA	Germany	2016 Nov 7	A/tufted_duck/Germany-SH/R8446/2016	Friedrich-Loeffler-Institut
EPI860239	HA	Denmark	2016 Nov 8	A/tufted duck/Denmark/17740–1/2016	Technical University of Denmark
EPI861572	HA	Croatia	2016 Nov 12	A/mute swan/Croatia/78/2016	Croatian Veterinary Institute
EPI861573	NA	Croatia	2016 Nov 12	A/mute swan/Croatia/78/2016	Croatian Veterinary Institute
EPI863857	HA	Sweden	2016 Nov 21	A/Chicken/Sweden/SVA161122KU0453/ SZ0209321/2016	National Veterinary Institute
EPI863859	NA	Sweden	2016 Nov 21	A/Chicken/Sweden/SVA161122KU0453/ SZ0209321/2016	National Veterinary Institute

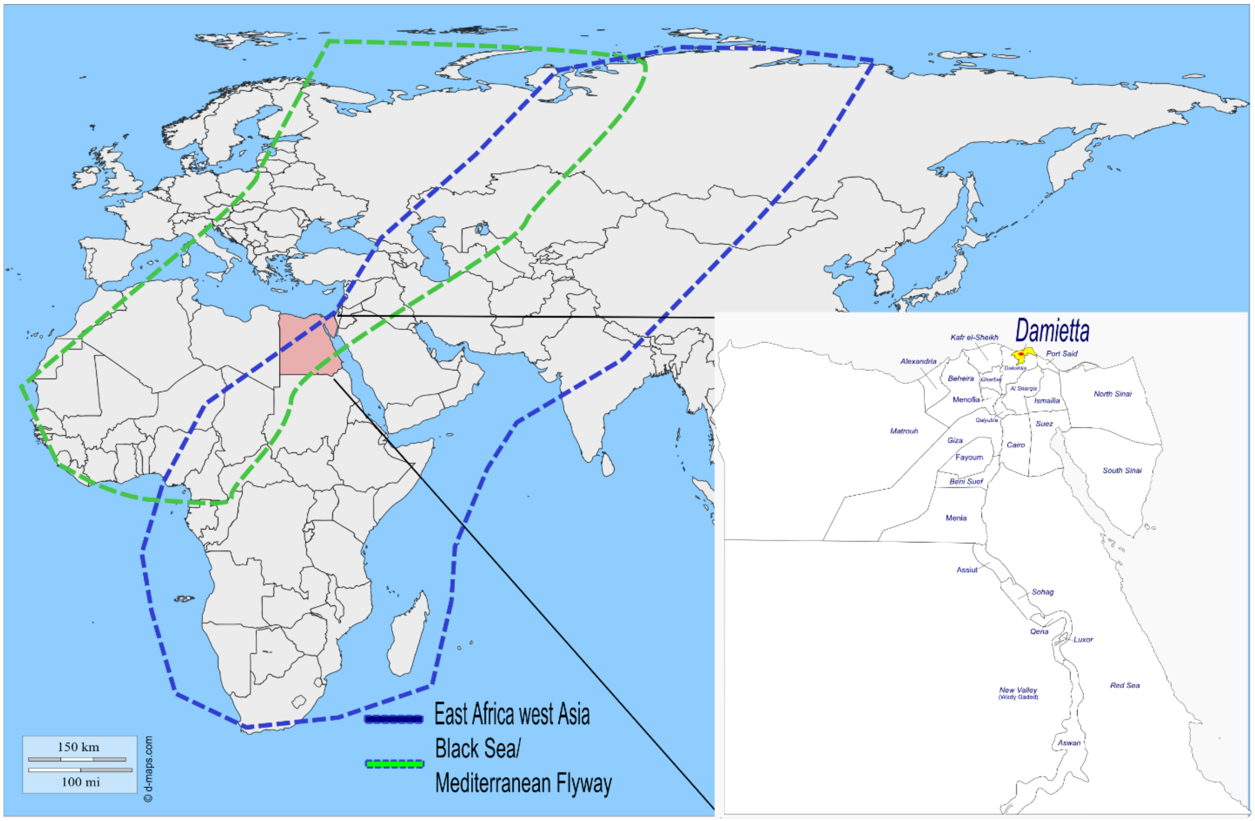

Technical Appendix Figure 1. Map showing various migratory flyways crossing Egypt. The area where highly pathogenic avian influenza virus, subtype H5N8, was detected in Damietta Governorate, Egypt, is indicated in yellow, and the infected live bird market is shown in red.

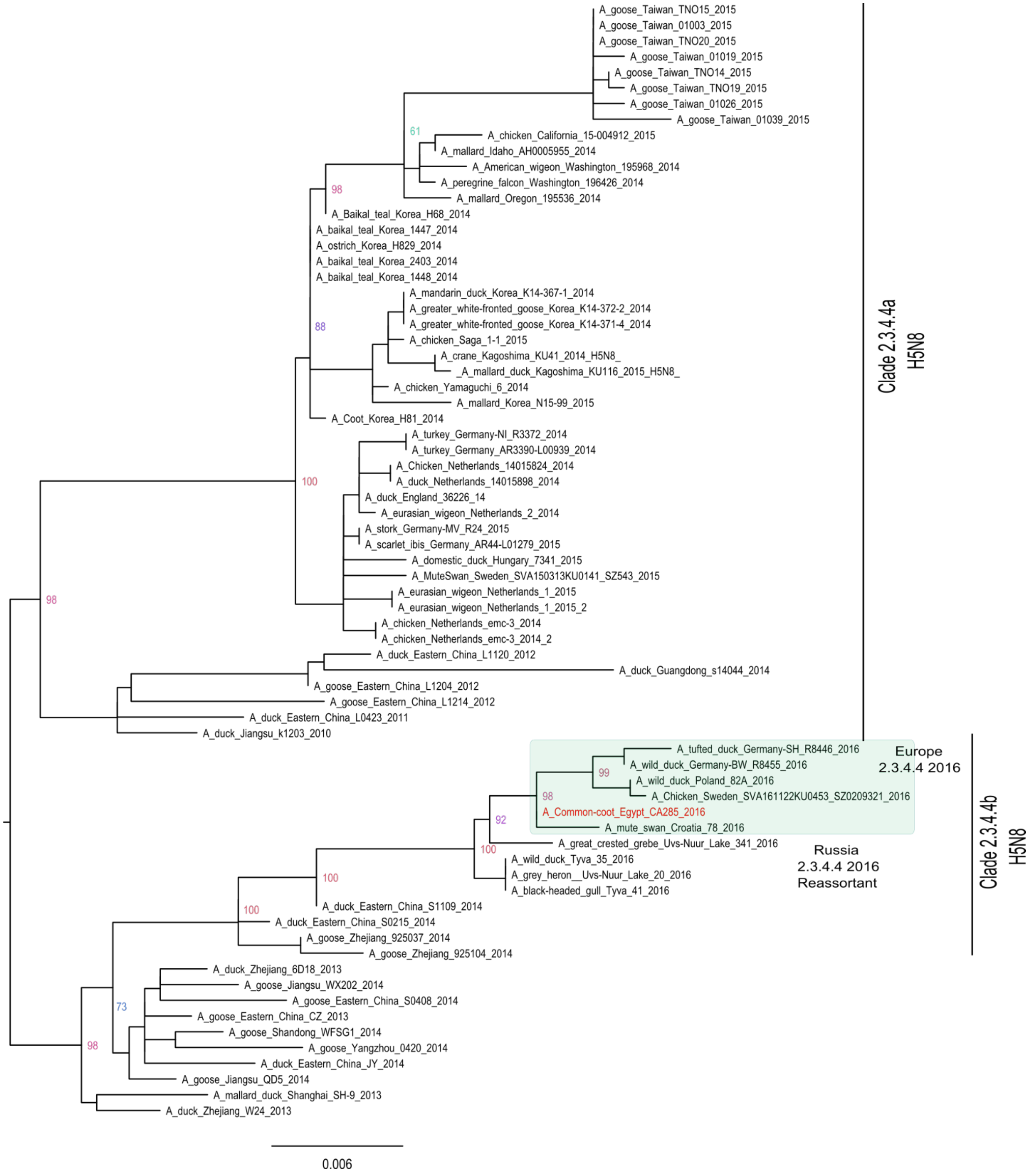

Technical Appendix Figure 2. Phylogenetic tree of the nucleotide sequences of the neuraminidase gene segments. Maximum likelihood calculations were done with the IQTree software under the best-fit model according to the Akaike criterion (HKY+G4 model). Highly pathogenic avian influenza virus strains (HPAIV), subtype H5N8, in Egypt are shown in red; current circulating HPAIV(H5N8) strains in Europe are highlighted in green.
